# The Sufferings of Christ

**Published:** 1887-11-05

**Authors:** 


### WORDS OF CONSOLATION.

[Communications for this column should be addressed "Chaplain," care of the Editor of THE HOSPITAL, who will gratefully welcome any suggestions or inquiries from matrons, nurses, and the staff generally.]

### LVII.—THE SUFFERINGS OF CHRIST.

(*For Reading to the Sick.*)

BODILY suffering is intense, or, on the contrary, very much moderated, in proportion to the sensitiveness of the nervous system. The more refined, and consequently the more perfect the nature physically, the more acutely pain is sometimes felt; whereas the more brutal animal natures amongst mankind certainly seem to suffer less acutely. Some people can bear pain much better than others. This may not always be because they are more courageous, but simply because they do not feel it so much. They have not the same kind of nervous system to be worked upon; they are, therefore, less afflicted by pain. Now, our Blessed Lord in his humiliation was perfect man, so perfect that, in spite of all He experienced in the way of toil and weariness and exhaustion, we never read of His being ill. His powers of endurance were evidently marvellous. But being so perfect and so refined in His human nature, this would make us think that He was very sensitive physically to pain. For this reason we differ widely from those who would diminish the severity of His bodily sufferings. We firmly believe that His physical pains were worse than most people have to endure, because His nature was so perfect; and, possibly, His nervous system so highly wrought. He can, therefore, say to those who suffer most in the flesh, "My own pains endured in the body were greater than thine."

It seems probable that sufferings hereafter will also proportion themselves according to the same law. The higher the nature bestowed by God, and then abused, degraded, and given over to sin, so according to law will be the retribution. Some who reject salvation will suffer more than others, simply from their own inherent capacity. "Many stripes" will not be inflicted as it were directly by the hand of God; rather they will be found to issue from the very nature of the sinner once so highly endowed, once so capable of the highest enjoyment, at last given over to reap in the body a retribution which must eventually be seen growing with terrible accuracy out of the seeds sown here. Different sins of the flesh (medical men know it well) produce seeds even here, from which various, but in many cases, *certain* results follow. Even the members of the body are observed to suffer according to their abuse, or the misapplication of their forces. Nature's law of retribution points this way. God's Word points this way. It is awfully instructive to observe how our Lord's passion points this way; in so far as it is an exhibition and manifestation of the pains sin could inflict upon One wholly innocent. He has not only offered a sacrifice in every member; but He warns us that if such things could be done in the green tree, worse, much worse, may be done in the dry (LUKE xxiii. 31). If so much suffering could be inflicted on Him who knew no sin, what will be the portion of the guilty when the day of salvation is past, and the springs of grace dry up for ever?

(*To be continued.*)

